# Antigen-armed antibodies against B-cell malignancies

**DOI:** 10.18632/oncotarget.26276

**Published:** 2018-11-02

**Authors:** Marta Ilecka, Dwain G. van Zyl, Henri-Jacques Delecluse

**Affiliations:** German Cancer Research Center (DKFZ), Unit F100, Heidelberg, Germany; Institut National de la Santé et de la Recherche Médicale (INSERM), Unit U1074, Heidelberg, Germany; German Center for Infection Research (DZIF), Braunschweig, Germany

**Keywords:** lymphoma, leukemia, immunotherapy, antigen-armed antibodies, CD4+ cytotoxic T cells

**Figure 1:**
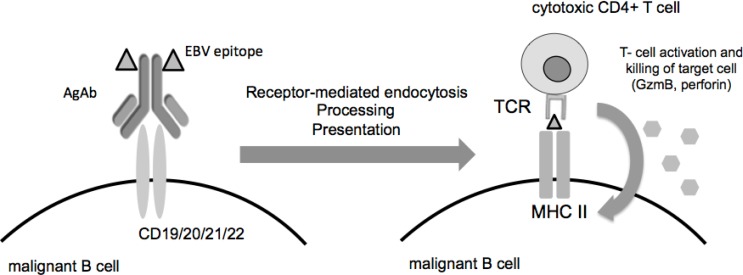
Schematic representation of AgAbs action An antibody conjugated to an EBV epitope recognizes its target on a malignant cell. Upon binding, the antibody gets internalized through receptor- mediated endocytosis. The antibody-epitope complex undergoes processing and the viral epitopes binding to MHC II molecules. This is followed by antigen presentation on the cell surface. The viral antigens in complex with the MHC II molecules are then available for recognition by cytotoxic CD4^+^ T cells.

Tremendous progress has been made in the field of cancer immunotherapy in the past few decades. The introduction of rituximab in 1997 marked a major milestone in the treatment of B-cell lymphomas and initiated the era of recombined anti-cancer antibodies. More recently, antibodies have been armed with cytotoxic drugs or radionuclides to increase their therapeutic potential or have been modified to enhance the immune response against tumor cells. The latter approach includes the development of bi-specific antibodies and of CAR-T cells that have since proven their efficacy in the clinic. Antibody-antigen conjugates represent another class of modified antibodies that were initially designed for vaccination purposes in various types of cells, including dendritic cells [1, 2]. Another branch of immunotherapy led to the development of antibodies against CTLA-4 and PD-1 to stimulate the T-cell response against tumor cells. This therapy is particularly efficient in tumors that express proteins with mutations or indels that render them immunogenic. We have recently repurposed antigen-armed antibodies (AgAbs) to redirect existing T-cell responses against common pathogens (e.g. viruses) towards tumor cells. The antibody moiety of the AgAbs specifically channels microbial antigens into the endosomal compartment of B cells after binding to the cell surface and internalization of the AgAb complex. As professional antigen- presenting cells, B cells possess cellular machinery that cleaves the immunogenic peptides contained in the AgAbs from the antibody carrier molecule and allows their presentation at the cell surface in complex with MHC II molecules (Figure 1). As a result, malignant B cells become eliminated by pathogen- specific CD4^+^ CTLs. Thus, this approach solves the requirement for mutated proteins to render the tumor cell visible to T cells. Although the choice of potential microbial antigens that can be inserted in AgAbs is practically unlimited, antigens derived from herpes viruses have multiple advantages. Epstein-Barr virus in particular latently infects 90% of the population, which guarantees the presence of EBV-specific T cells in a large proportion of cancer patients. Furthermore, the lifelong persistence of the virus in infected individuals guarantees that these T cells are present in substantial numbers at any age.

We previously demonstrated that AgAbs directed against CD19/CD20/CD21/CD22 combined to HLA II-restricted epitopes encoded by the Epstein-Barr virus genome can eliminate multiple Burkitt's lymphoma cell lines *in vitro*. These AgAbs were efficiently internalized and processed, leading to the effective presentation of epitopes on the surface of tumor cells and resulted in efficient T-cell mediated killing. The recognition and killing rates were far superior to the response elicited by the same EBV peptides in stoichiometric proportions [3]. As expected by the very high rate of EBV infection in the general population, AgAbs stimulated the expansion and activation of EBV-specific memory T cells from seropositive individuals *ex vivo.* The versatility of the AgAb system allows the incorporation of multiple EBV epitopes that elicit a T-cell response in the majority of EBV-infected individuals. We have recently shown that this approach is feasible and efficient for patients with chronic lymphocytic leukemia (CLL). AgAbs carrying multiple epitopes from the strongly immunogenic EBNA3C protein successfully expanded EBNA3C-specific CD4+ T cells in 12 blood samples from CLL patients [4]. In all samples, exposure of CLL cells to EBNA3C AgAbs led to epitope presentation and high rates of T cell killing, reaching up to 80% is some cases. This result was quite unexpected as this incurable disease is characterized by impaired T-cell responses in the majority of patients, as well as by low CD80 and CD86 expression in the tumor cells [5]. Nonetheless, virus-specific T cells seem to remain functional in CLL patients and stimulation with EBNA3C AgAbs led to a powerful antitumor T cell response.

These results offer another example of the cytotoxic potential of CD4+ cells that share typical gene expression characteristics with CD8+ T cells and whose differentiation is dependent on CRTAM expression [6]. CD4+ CTLs play a crucial role in the defense against multiple viruses, including the Epstein- Barr virus (EBV), human cytomegalovirus or Dengue virus [7-9]. Moreover, tumor- specific CD4+ T cells are able to eliminate malignant cells in a melanoma model [10]. These reports clearly prove that CD4+ T cells can develop cytotoxic activity and play an important role in immunity against viruses and cancer that is currently underestimated.

In summary, antigen-armed antibodies represent a new class of immunostimulatory molecules with a great potential in cancer immunotherapy. A great advantage of the strategy is its flexibility as antibodies recognizing a broad spectrum of targets can be easily created, tailoring the therapy to changes and differences in antigens expression. The application of AgAb strategy can extend beyond lymphoid malignancies, provided that they can be tagged by antibodies and express MHC class II molecules. The potential of these molecules is currently being tested in animal models before the inception of clinical trials.
